# Preliminary Results of a Bicycle Training Course on Adults’ Environmental Perceptions and Their Mode of Commuting

**DOI:** 10.3390/ijerph19063448

**Published:** 2022-03-15

**Authors:** Patricia Gálvez-Fernández, Palma Chillón, María Jesús Aranda-Balboa, Manuel Herrador-Colmenero

**Affiliations:** 1 PROFITH “PROmoting FITness and Health through Physical Activity” Research Group, Sport and Health University Research Institute (iMUDS), Department of Physical Education and Sports, Faculty of Sport Sciences, University of Granada, 18071 Granada, Spain; pgalvez@ugr.es (P.G.-F.); mjab@ugr.es (M.J.A.-B.); mhc@ugr.es (M.H.-C.); 2 La Inmaculada Teacher Training Centre, University of Granada, 18013 Granada, Spain

**Keywords:** active transportation, bicycle training skills course, Bikeability, mode of travel, perceptions, environment, public health

## Abstract

This study was designed to analyze the effects of a bicycle training course on both adults’ environmental perceptions and their mode of commuting. Four bicycle training courses for adults were conducted in Granada, Spain in April 2015 and May 2016. The course program was focused on developing practical skills and attitudes on road. From the initial 65 adults who started the course, only 35 adults met the inclusion criteria and were included in the study. Participants completed twice (i.e., before and after the course) a questionnaire about their perceptions of the environment, usual mode of commuting to daily destinations, and sociodemographic characteristics. After finishing the initial questionnaire, the participants completed a bicycle training course based on the methodology “Bikeability” with a duration of 6 h. The results suggest that participants improved their safety perception in relation to the level of crime in the participants’ neighborhood after the bicycle training course. Cycling training courses should last longer in order to produce changes in the mode of commuting and in the environmental perceptions.

## 1. Introduction

Regular and sustained participation in physical activity is linked to reduced chances of acquiring numerous health conditions in adults [1,2,3]. However, a third or more of adults worldwide do not meet the minimum of 150 min of moderate-to-vigorous physical activity [4] recommended by the World Health Organization [5]. In fact, there is a widespread concern about the low level of physical activity in the general population [6]. This fact contributes to a growing incidence of problems such as obesity and chronic diseases, which are increasing in Spain and Europe [7].

Active commuting is a behavior that implies the performance of physical activity using ecological and non-motorized modes to move to a destination. A daily active commuting behavior is moving from/to home and workplace/school [8]. Active commuting has been associated with a higher level of total physical activity in adults [9,10]. In addition, active commuting has several health implications for adults such as obesity prevention [11] and increased muscle strength [12]. Compared to walking, cycling is associated with risk reduction in cardiovascular diseases, diabetes, and cancer [13], and with improvement in mental health [14]. In addition, cycling also has environment and economic benefits [15].

Consequently, in order to achieve the physical activity recommendations, adults might choose to bicycle from home to their habitual destinations [16]. However, the limited evidence in Spain shows very low levels of cycling in adult population. Indeed, only around 11% of young adults, aged between 18–29 years old, bicycle to university [17]. Moreover, around 8% and 10% of adults, aged between 25–39 years old, bicycle to usual destinations and work every day, respectively [18], while 2.5% of healthy women, aged 37–65 years old, bicycle to the supermarket [19].

Since cycling in a safe manner requires developing sufficient motor and cognitive abilities [20], bicycle training courses are becoming popular, with a wide variety of methodologies to teach cycling in the urban context [21]. Given that the main objective is to acquire practical skills and understanding on cycling on the road, the contents of the bicycle training methodologies focus on: knowledge about road safety rules, safety equipment, and hand signals for cyclists; skills related to cycling in an urban context without traffic and on the road; and attitudes related to awareness, confidence, enjoyment, and usefulness of cycling [22,23,24,25]. Examples of bicycle training courses are “Bikeability” [22,23] and “Cycle for Health” [24] which are widely used in the United Kingdom, and “Bike Smart” [25] in the United States. Concretely, Goodman et al. [22], who implemented the Bikeability methodology, observed short-term improvements in bicycle management and control in children after participating in bicycle training courses in the urban context. Additionally, Goodman et al. [22] managed to modify the perception of the participants of becoming more independent in the use of the bicycle. McLaughlin and Glang [25], who implemented the Bike Smart methodology, also found improvements in the identification and application of cycling safety techniques in children.

In the scientific literature, several studies have analyzed the effects of the bicycle training courses in the urban context for adults settled in Australia [26,27] and the United Kingdom [28]. Among the Australian studies, Telfer et al. [26] observed an increase in the frequency of trips by bicycle and an improvement in basic skills and confidence to ride the bicycle after the participation of adults in the cycling program. Devine et al. [27] reported an improvement in social relationships and an increase of cycling trips. Moreover, in terms of changes in cycling levels, similar results were found in the United Kingdom, where Johnson et al. [28] reported higher cycling frequency after the training than beforehand. As for Spain, there are gaps in the bicycle training courses carried out in cities, such as the Biciclot method in Terrassa. The aforementioned course is focused on checking the bicycle before cycling and road safety rules for cycling. Another course is the Aula Bici methodology in Valencia, that carries out practical sessions in the neighborhood’s grounds to learn or improve the fundamental cycling skills needed to ride the bicycle safely. There are no specific studies providing data on the effectiveness of these methodologies yet in adults. We posit that bicycle training courses have great effects on environmental perceptions and active modes of commuting in adults.

Research on rates of cycling across countries is highly determined by certain con-textual elements [29], such as the urban environment where the population lives [30]. Urban environment is an important contextual factor that differs in each country, as well as government policies, programs to promote cycling, and extensive cycling support infrastructures [31].

Cycling behavior may be influenced by the environmental perceptions in adults [32]. Actually, traffic safety [33,34], the residential density of the area [35], the length of the route [36], and the scarcity of bicycle parking places [37] are the most highlighted negative environmental perceptions that inhibit cycling behavior in adults. Moreover, several perceptions of safety and infrastructure are largely influenced by individual factors such as gender, age, and experience of cycling [38]. Therefore, studies are warranted in order to have evidence about the environmental perceptions in Spain and their influence on cycling behavior within urban contexts.

Regarding the recent changes of the current society into the environmental paradigm, whose worldwide framework is currently the Sustainable Development Goals (SDG) 2030 addressed by the United Nations [39], more evidence on the effects of bicycle training courses for adults is lacking. Therefore, there is a clear need to review the effects of bicycle training course in adults in order to develop an effective course to change the mode of commuting and to improve the environmental perceptions. The aim of the current study was to analyze the effect of bicycle training on the environmental perceptions and mode of commuting among Spanish adults.

## 2. Materials and Methods

### 2.1. Study Design and Participants

Four bicycle training courses implemented in April 2015 and May 2016 (two each year) were conducted in Granada, Spain. The cycling training courses are a part of the strategy of the city council of Granada to promote the bicycle as a mean of transport. It was announced to the population through promotional flyers, email, posters, and media releases. The courses focused on developing practical skills and attitudes on the road. They were free and voluntary courses for beginner and intermediate level urban cyclists, comprising 6 h of duration. The maximum number of participants allowed per course was eighteen, with an instructor for every six participants. Participants were selected following the order of registration.

A total of 65 adults from Granada (Spain) participated freely in a bicycle training course. Participants met with the project coordinator who explained the nature of the study. They also completed a questionnaire on two occasions: (i) written on paper, 15 min before taking part in the course, and (ii) online, 15 days after finishing the course. To be included in the study, participants had to provide (a) valid questionnaire data on mode of commuting and perceptions of the environment at the two measurement times, and (b) data about age, gender, and socioeconomic status.

The anonymity of the research participants was preserved at all times, as stated in the ethical considerations of Sport and Exercise Science Research [40] and the principles included in the Declaration of Helsinki [41], which defines the ethical guidelines for research involving human subjects. In addition, the present study has been approved by the Ethical Committee of experimentation of the University of Granada (case no. 356). Written informed consent was filled by the participants and collected. Similarly, throughout the intervention and thereafter, we acted in accordance with the Spanish regulations established in the Organic Law 15/1999, of 13 December, regarding the protection of personal data.

### 2.2. Measures

Participants were asked to complete a questionnaire on their perceptions of the environment [42], mode of commuting to usual destinations [43], and socioeconomic data [44].

#### 2.2.1. Environmental Perceptions

The environmental perceptions of the neighborhood where the participants lived was assessed using the short version of the Assessing Levels of Physical Activity and Fitness (ALPHA) questionnaire [42]. This questionnaire has been previously validated in adults aged 20 to 65 years old. The Spanish version has been used before, in spite of being only validated for Spanish adolescents aged 12 to 18 years old [45]. The questionnaire comprises 10 items with a Likert scale as response options (see Supplementary Table S1).

#### 2.2.2. Modes of Commuting

The mode of commuting was assessed using five questions and seven response options (see Supplementary Table S2) in relation to their usual destinations (i.e., to local shops, to the supermarket, and to local services) and to and from work, given the reliability of these questions in a study performed in Spanish women with fibromyalgia [43].

#### 2.2.3. Socioeconomic Data

Socioeconomic data were assessed through the following questions: “Which is the maximum level of studies that you have?” (Response options: no studies, primary education, compulsory secondary education, upper secondary education, professional training, and university studies); and “Indicate your work activity” (response options: do not work and work). Socioeconomic data were adapted from the 2006 National Health Survey [46].

### 2.3. Intervention: Cycle Training Course in the Urban Context

The bicycle training course in the urban context took place for 6 h. This course was an adaptation of the Bikeability method [23]. An active and participatory methodology was used through the assignment of tasks and cognitive-teaching activities, that were simple actions that participants have to perform when cycling in the urban context. In this way, the knowledge and skills programmed were previously discussed among the participants to reinforce positive aspects and help them to solve negative aspects.

**Figure 1:**
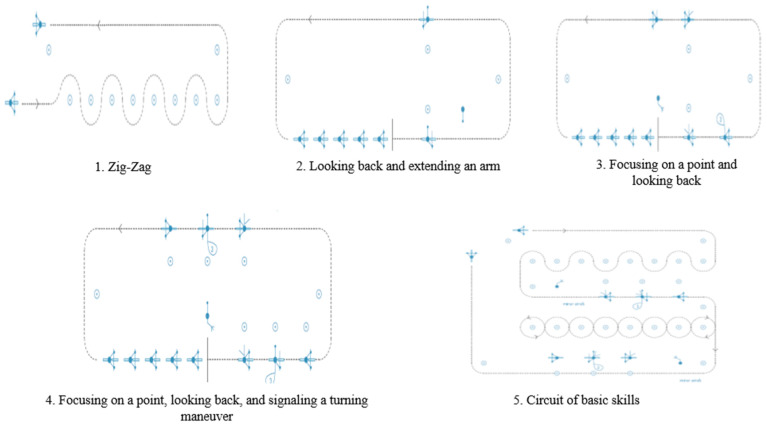
Exercises in the closed traffic circuit.

**Figure 2:**
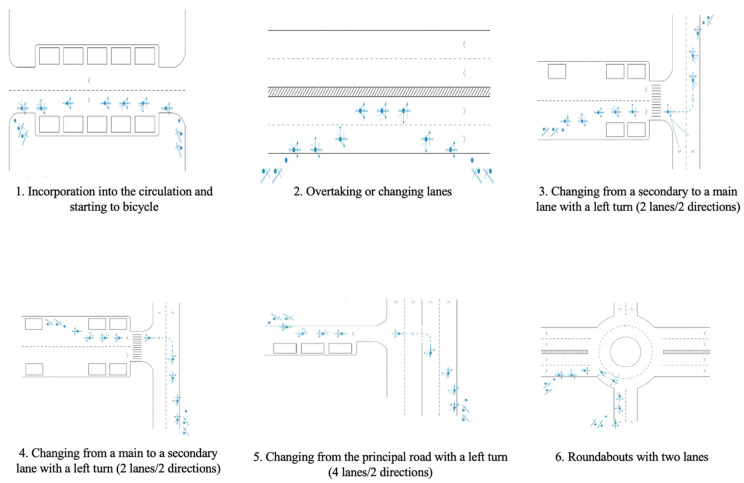
Exercises in the urban circuit.

The bicycle training course has been previously described [47]. Briefly, it comprised three parts: (a) an awareness-raising session on the use of the bicycle in the city (1 h), in which participants became aware of the advantages and disadvantages of cycling and road traffic regulations; (b) a session in a closed traffic circuit and a 15-min break (2 h), with the aim of developing basic skills for handling and controlling the bicycle (Figure 1); and (c) a session in an urban circuit, in real traffic conditions (3 h), where they applied the learned skills for riding the bicycle to a real context (Figure 2).

### 2.4. Statistical Analyses

Firstly, the Shapiro–Wilk test (*n* < 50) was used to assess the normality of data. As normal distribution was not found in our data, non-parametric tests were used. Age, gender, and sociodemographic characteristics of the participants were analyzed using descriptive statistics. The data are presented as mean ± standard deviation for continuous variables and frequencies for categorical variables. The variable mode of commuting was recoded into a three categories multinomial variable: walking, cycling, and passive (i.e., by car, by motorcycle, by bus, or by subway/train) and into three dichotomous variables: walking vs. not walking, cycling vs. not cycling, and active (i.e., walking or cycling) vs. passive. To analyze the differences before and after the course in the environmental perceptions of the area in their neighborhood, we used the Wilcoxon signed-rank test. To determine age invariance, we used median as a criterion to create two groups. Effect sizes were assessed with the Pearson´s correlation (r) and Cramer’s V for continuous and categorical variables, respectively. Effect sizes were considered small, moderate, or large, when (r) was above 0.30, between 0.31 and 0.49, and 0.50 [48], respectively, and when Cramer’s V was above 0.10, 0.30, and 0.50, respectively [49]. We used the McNemar test to analyze the differences in the mode of commuting before and after the bicycle training course in the urban context. All analyses were carried out through the IBM SPSS Statistics 22.0 statistical package with a significance level of *p* < 0.05.

## 3. Results

From the initial sample, 46% of participants did not meet the inclusion criteria because they did not complete questionnaires in the pre-intervention and/or post-intervention. A total of 35 participants (54% women) met the inclusion criteria and were included in this study. The average age of the participants was 34 years old (age range: 19 to 53 years old) and 74% were workers.

The different response options for each question of the environmental perceptions before and after taking part in the bicycle training course in the urban context are described in Table 1. A significant difference in the safety perception in relation to the level of crime in the participant’s neighborhood, which was lower in pre-intervention (mean = 9.67) than in post-intervention (mean = 11.53) (*p* < 0.05), was found. In contrast, no significant differences were found for the other environmental perception variables (all, *p* > 0.05).

Table 2 indicates the results concerning the intra-group analysis for gender and age groups. In this sense, the Wilcoxon Signed-Rank test revealed that no significant statistical difference (*p* > 0.05) was found for each question of the environmental perceptions before and after taking part in the bicycle training course in the urban context between the pre– and the post-intervention.

The numbers of trips using different modes of commuting before and after taking part in the bicycle training course in the urban context are shown in Table 3. Analyzing all modes of commuting together, significant differences in the mode of commuting used to local shops and local services were found (all, *p* < 0.05). When the analysis was performed for each mode of commuting, significant differences were found in the mode of commuting to local shops and to local services, with a reduction in the walking trips (Difference: *n* = −15, *p* = 0.001; Difference: *n* = −10, *p* = 0.013, respectively) and an increase in passive trips (Difference *n* = 13, *p* = 0.004, Difference *n* = 12, *p* = 0.002, respectively). For cycling trips, no significant differences were found (all *p* > 0.05).

## 4. Discussion

In the present study, a bicycle training course was implemented to examine the changes in some environmental perceptions and the mode of commuting of Spanish young adults. Concerning the environmental perceptions, we found significant differences in safety perception regarding crime, which improved after the bicycle training course. Regarding the mode of commuting, we observed a significant decrease in walking trips and an increase in passive trips to local services and local shops after the course. Regarding cycling, there were no significant improvements.

The results obtained in this study showed that the safety perception in relation to the level of crime in the participant’s neighborhood was improved after the bicycle training course. In the scientific literature, there are no studies reporting improvements in the safety perception in relation to crime after a course in adults. However, there are various studies in adults that closely correlated with confidence gained from the training [50,51]. Rissel and Watkins [50] and Zander et al. [51] showed how adults improved their confidence cycling on the road or with traffic after the course. These results concur in part with those of the present study, since it has been verified that the improvement in the safety perception in relation to the level of crime in the participant’s neighborhood is associated with an improvement in confidence related to safety perception cycling on the road [28]. This association could be due to several reasons. First, an increase in walking and cycling trips around the neighborhood may generate more contact and knowledge of the urban environment. Consequently, it is more likely to feel more confident than when using motorized modes [52]. Second, behavioral changes in the attitude of participants related to their perception of crime in the environment could be possible because an improvement perception of the environment may encourage an increase in exercise [53]. Third, since the bicycle training course was performed on a road with real traffic and not in a neighborhood, perhaps this context could increase their confidence on the bicycle and consequently improve their perception of crime. Thus, it is important to develop interventions to improve environmental perceptions and cycling trips in adults. Moreover, given the importance of the neighborhood environment, it is required that policy makers improve the urban context. For example, building safe bicycle lanes and/or other safety bicycle infrastructures may increase the confidence and safety of cyclists.

After the intervention, our results showed a significant decrease in walking trips and an increase in passive trips to local services and local shops. In the scientific literature, few studies have addressed the effect of bicycle training courses on the urban context among the adult population. Indeed, to our knowledge, only one study reported the effects of the bicycle training courses in the urban context for adults [26]. Attending to all modes of commuting together, the previous study suggested that the mode of commuting to usual destinations can be modified after the implementation of a bicycle training course in adults [26]. Specifically, Telfer et al. [26] showed an increase in the number of cycling trips in Australian adults, although this was not statistically significant. Regarding cycling, the previous study showed similar results to our study, where cycling trips to the above-mentioned destinations increased, but not significantly. The decrease in walking trips and increase in passive trips observed in the present study in adults could be due to different reasons. First, changing mobility habits in a short period of time could be complex [54]. The intervention of the present study had a short period of time (six hours), and perhaps participants need a longer period of adaptation to change their mode of commuting. According to Telfer et al. [26], Australian participants answered the post-intervention questionnaire after two months, which is enough time to improve the learned skills in the bicycle training course and to ride on a bicycle to usual destinations. Conversely, in the present study, Spanish participants answered the post-intervention questionnaire after only 15 days. Second, the presence of slopes in the terrain orography could be an important factor in the decrease in active modes to usual destinations. Indeed, a negative association has been found between slopes and active commuting [55]. Perhaps, it could be speculated that Australian studies obtained better improvements due to the flat geography and bike-friendly infrastructure of Sydney compared to Granada, which has numerous slopes. Third, the mode of commuting could change every day [56]. So, participants may not have an established commuting routine and depending on their schedule in the study period, they could answer one thing or another. Fourth, the origin and “bicycle culture” of the participants or attitudes and meanings toward using bicycles could modify mobility habits [57]. Fifth, the distance to usual destinations could produce these results [34]. Perhaps participants could use their bicycles only for short trips and not for usual destinations, until achieving confidence and security on the bicycle. Finally, other factors that could be important for changing the mode of commuting have not been evaluated in this study, such as, climatic conditions [58]. Future studies should attend to the rates of modes of commuting observed in their countries and analyze the potential reasons for their increasing or decreasing, to ensure the efficiency of bicycle training courses, since the environmental awareness of society seems to be improving.

The main strength of this study is to provide the first Spanish study about how to implement and analyze the effectiveness of a bicycle training course for adults in the urban context. In addition, a recognized methodology (i.e., “Bikeability”) was used to teach bicycle training courses, similar to other previous experiences in other countries. The main limitation is a lack of representative data at the national level because the study was restricted to a single geographical area, the city of Granada, in southern Spain. Moreover, the use of the Spanish version of the ALPHA questionnaire and the questions on mode of commuting for adults is another limitation of this study, since it has not been shown to be valid and reliable in healthy adults. However, the ALPHA questionnaire and the mode of commuting questions are reliable, and they have been associated with physical activity in women with fibromyalgia. In addition, the sample size is low, which hampers the application of powerful statistical analyses. Finally, other limitations are the lack of individual neighborhood data, weather conditions, the lack of motivations or concerns of the participants to take the course, and objective measures to collect data on modes of commuting.

## 5. Conclusions

The present study showed how a bicycle training course can improve safety perceptions in relation to the level of crime in the participants’ neighborhood. However, a decrease in walking trips and increase in passive trips were found. In contrast, no significant results in cycling trips were observed. After this experience, we have concluded that changing commuting habits may require longer bicycle training courses over time and/or further follow-up measurements to assess the long-term effects of these cycling training interventions. Moreover, it is important that bicycle training courses focus on enhancing adult people’s cycling confidence by improving skills, practicing, and repeating.

## Figures and Tables

**Table 1:** Cycle training course effects on adults’ perceptions of the environment.

Perceptions of Environment (Items)	N	Test	Mean Rank	Sum of Ranks	Z	*p*-Value	r
Most of the houses in my neighborhood are detached houses	34	Pre	3.50	10.50	−0.632	0.527	0.10
		Post	4.38	17.50			
Many shops, stores, markets, or other places to buy things I need are within easy walking distance of my home	34	Pre	4.83	14.50	−0.513	0.608	0.08
		Post	4.30	21.50			
There is a transit stop (such as bus stop, train, trolley, or tram station) within easy walking distance of my home away	34	Pre	3.20	16.00	−1.171	0.238	0.20
		Post	5.00	5.00			
There are many different routes for cycling or walking from place to place in my neighborhood so I don’t have to go the same way every time	34	Pre	6.67	40.00	−0.821	0.412	0.14
		Post	5.13	65.00			
Walking and cycling are unsafe because of the traffic in my neighborhood	34	Pre	12.13	134.00	−0.696	0.486	0.12
		Post	10.31	97.00			
Walking and cycling are safe because of the level of crime in my neighborhood	33	Pre	9.67	58.00	−2.070	0.039	0.36
		Post	11.53	173.00			
My local neighborhood is a pleasant environment for walking and cycling	34	Pre	9.21	64.50	−1.559	0.119	0.26
		Post	11.19	145.50			
There is an open recreation area (e.g., park, beach, or other open space) within easy walking distance of my home	34	Pre	10.14	71.00	−0.656	0.512	0.11
		Post	9.09	100.00			
I have access to exercise and sport facilities at work e.g., fitness center/equipment, stairs	34	Pre	8.79	74.50	−0.341	0.733	0.05
		Post	8.28	61.50			
My work place provides facilities to support me walking or cycling to work e.g., changing rooms, bike storage	33	Pre	6.00	37.00	−0.978	0.328	0.17
		Post	5.29	18.00			

**Table 2:** Intra-Group Analysis Through the Wilcoxon Signed-Rank Test for bicycle training course effects on adults’ perceptions of the environment.

		Gender							Age					
Perceptions of Environment (Items)	*n*	Group	Mean Rank	Sum of Ranks	Z	*p*-Value	r	*n*	Group	Mean Rank	Sum of Ranks	Z	*p*-Value	r
Most of the houses in my neighborhood are detached houses	15	M	2.00	2.00	−0.577	0.564	0.10	16	Y	1.00	1.00	−1.000	0.317	0.17
	19	F	2.00	4.00				18	O	0.00	0.00			
Many shops, stores, markets, or other places to buy things I need are within easy walking distance of my home	15	M	2.00	4.00	−0.577	0.180	0.09	16	Y	2.50	5.00	<0.001	1.000	0.01
	19	F	2.00	2.00				18	O	2.50	5.00			
There is a transit stop (such as bus stop, train, trolley, or tram station) within easy walking distance of my home away	15	M	1.50	3.00	−1.342	0.564	0.23	16	Y	1.50	3.00	−1.342	0.180	0.23
	19	F	0.00	0.00				18	O	0.00	0.00			
There are many different routes for cycling or walking from place to place in my neighborhood so I don’t have to go the same way every time	15	M	2.00	2.00	−0.577	0.564	0.14	16	Y	2.00	4.00	−0.378	0.705	0.06
	19	F	2.00	4.00				18	O	3.00	6.00			
Walking and cycling are unsafe because of the traffic in my neighborhood	15	M	4.00	20.00	−0.312	0.755	0.12	16	Y	4.50	22.50	<0.001	1.000	0.01
	19	F	6.25	25.00				18	O	5.63	22.50			
Walking and cycling are safe because of the level of crime in my neighborhood	15	M	3.75	7.50	−1.496	0.135	0.26	16	Y	6.00	12.00	−0.905	0.366	0.16
	18	F	4.75	28.50				17	O	4.00	24.00			
My local neighborhood is a pleasant environment for walking and cycling	15	M	3.50	10.50	−1.469	0.142	0.26	16	Y	4.38	17.50	−0.632	0.527	0.11
	19	F	5.75	34.50				18	O	3.50	10.50			
There is an open recreation area (e.g., park, beach, or other open space) within easy walking distance of my home	15	M	3.50	7.00	−1.265	0.206	0.21	16	Y	5.63	22.50	<0.001	1.000	0.01
	19	F	4.20	21.00				18	O	4.50	22.50			
I have access to exercise and sport facilities at work e.g., fitness center/equipment, stairs	15	M	3.60	18.00	−1.581	0.114	0.27	16	Y	4.60	23.00	−0.705	0.481	0.12
	19	F	3.00	3.00				18	O	4.33	13.00			
My work place provides facilities to support me walking or cycling to work e.g., changing rooms, bike storage	15	M	3.80	19.00	−1.807	0.071	0.31	16	Y	3.63	14.50	−0.085	0.932	0.01
	18	F	2.00	2.00				17	O	4.50	13.50			

Notes M = Male; F = Female; Y = Younger adults; O = Older adults.

**Table 3:** Cycle training course effects on adults’ mode of commuting.

		Modes of Commuting (Walk/Bike/Passive)			*p*-Values					
	*n*	Pre (*n*)	Post (*n*)	Difference (Post-Pre)	x2 (df)	V	Total	Walk	Bike	Passive
Commuting to local shops	35	25/6/4	10/8/17	−15/2/13	11.8 (3)	0.410	0.019	0.001	0.687	0.004
Commuting to the supermarket	36	22/3/11	14/8/14	−8/5/3	6.3 (3)	0.295	0.096	0.077	0.375	0.388
Commuting to local services	36	21/8/7	11/6/19	−10/−2/12	10.7 (3)	0.385	0.013	0.013	0.687	0.002
Commuting home–work place	22	4/9/9	2/4/16	−2/−5/7	5.4 (3)	0.350	0.141	0.687	0.125	0.065
Commuting work place–home	22	4/8/10	3/3/16	−1/−5/6	4.3 (3)	0.312	0.228	1.000	0.125	0.146

## Data Availability

Not applicable.

## Supplementary Materials

The following supporting information can be downloaded at: https://www.mdpi.com/article/10.3390/ijerph19063448/s1, Table S1. Short version of the Assessing Levels of Physical Activity and fitness (ALPHA) questionnaire; Table S2. Mode of commuting questions.

## References

[1] Liu-Ambrose T., Barha C., Falck R.S. (2019). Active body, healthy brain: Exercise for healthy cognitive aging. Int. Rev. Neurobiol..

[2] Martínez-Gomez D., Lavie C.J., Hamer M., Cabanas-Sanchez V., Garcia-Esquinas E., Pareja-Galeano H., Struijk E., Sadarangani K.P., Ortega F.B., Rodríguez-Artalejo F. (2019). Physical activity without weight loss reduces the development of cardiovascular disease risk factors—A prospective cohort study of more than one hundred thousand adults. Prog. Cardiovasc. Dis..

[3] Rhodes R.E., Janssen I., Bredin S.S.D., Warburton D.E.R., Bauman A. (2017). Physical activity: Health impact, prevalence, correlates and interventions. Psychol. Health.

[4] Hallal P.C., Andersen L.B., Bull F.C., Guthold R., Haskell W., Ekelund U. (2012). Global physical activity levels: Surveillance progress, pitfalls, and prospects. Lancet.

[5] Bull F.C., Al-Ansari S.S., Biddle S., Borodulin K., Buman M.P., Cardon G., Carty C., Chaput J.P., Chastin S., Chou R. (2020). World Health Organization 2020 guidelines on physical activity and sedentary behaviour. Br. J. Sports Med..

[6] Ramos P., Jiménez-Iglesias A., Rivera F., Moreno C. (2016). Physical Activity Trends in Spanish Adolescents. Rev. Int. Med. Cienc. Act. Física Deporte.

[7] Bassett D.R., Pucher J., Buehler R., Thompson D.L., Crouter S.E. (2008). Walking, cycling, and obesity rates in Europe, North America, and Australia. J. Phys. Act. Health.

[8] Henriques-Neto D., Peralta M., Garradas S., Pelegrini A., Pinto A.A., Sánchez-Miguel P.A., Marques A. (2020). Active Commuting and Physical Fitness: A Systematic Review. Int. J. Environ. Res. Public Health.

[9] Berglund E., Lytsy P., Westerling R. (2016). Active Traveling and Its Associations with Self-Rated Health, BMI and Physical Activity: A Comparative Study in the Adult Swedish Population. Int. J. Environ. Res. Public. Health.

[10] Rissel C., Mulley C., Ding D. (2013). Travel mode and physical activity at Sydney University. Int. J. Environ. Res. Public. Health.

[11] Mytton O.T., Panter J., Ogilvie D. (2016). Longitudinal associations of active commuting with body mass index. Prev. Med..

[12] Larouche R., Faulkner G., Tremblay M.S. (2016). Active travel and adults’ health: The 2007-to-2011 Canadian Health Measures Surveys. Health Rep..

[13] Celis-Morales C.A., Lyall D.M., Welsh P., Anderson J., Steell L., Guo Y., Maldonado R., Mackay D.F., Pell J.P., Sattar N. (2017). Association between active commuting and incident cardiovascular disease, cancer, and mortality: Prospective cohort study. BMJ.

[14] Mueller N., Rojas-Rueda D., Salmon M., Martinez D., Ambros A., Brand C., De Nazelle A., Dons E., Gaupp-Berghausen M., Gerike R. (2018). Health impact assessment of cycling network expansions in European cities. Prev. Med..

[15] Gössling S., Choi A., Dekker K., Metzler D. (2019). The social cost of automobility, cycling and walking in the European Union. Ecol. Econ..

[16] Del Duca G.F., Nahas M.V., Garcia L.M., Silva S.G., Hallal P.C., Peres M.A. (2016). Active commuting reduces sociodemographic differences in adherence to recommendations derived from leisure-time physical activity among Brazilian adults. Public Health.

[17] Molina-Garcia J., Sallis J.F., Castillo I. (2014). Active commuting and sociodemographic factors among university students in Spain. J. Phys. Act. Health.

[18] Barómetro de la Bicicleta. https://www.ciudadesporlabicicleta.org/wp-content/uploads/2019/12/RCxB-Barómetro-de-la-Bicicleta-2019.pdf.

[19] Herrador-Colmenero M., Álvarez-Gallardo I.C., Segura-Jiménez V., Estévez-López F., Soriano-Maldonado A., Ruiz-Montero P.J., Tercedor P., Girela-Rejón M.J., Delgado-Fernández M., Chillón P. (2016). Associations between patterns of active commuting and socioeconomic factors in women with fibromyalgia: The al-Andalus project. Clin. Exp. Rheumatol..

[20] Briem V., Radeborg K., Salo I., Bengtsson H. (2004). Developmental aspects of children’s behavior and safety while cycling. J. Ped. Psych..

[21] Mölenberg F.J., Panter J., Burdorf A., van Lenthe F.J. (2019). A systematic review of the effect of infrastructural interventions to promote cycling: Strengthening causal inference from observational data. Int. J. Behav. Nutr. Phys. Act..

[22] Goodman A., van Sluijs E.M., Ogilvie D. (2016). Impact of offering cycle training in schools upon cycling behaviour: A natural experimental study. Int. J. Behav. Nutr. Phys. Act..

[23] Department for Transport, UK (2015). Bikeability. https://bikeability.org.uk/.

[24] Lovelace R., Beck S., Watson M., Wild A. (2011). Assessing the energy implications of replacing car trips with bicycle trips in Sheffield, UK. Energy Policy.

[25] McLaughlin K.A., Glang A. (2010). The effectiveness of a bicycle safety program for improving safety-related knowledge and behavior in young elementary students. J. Pediatr. Psychol..

[26] Telfer B., Rissel C., Bindon J., Bosch T. (2006). Encouraging cycling through a pilot cycling proficiency training program among adults in central Sydney. J. Sci. Med. Sport.

[27] Devine E., Handmer M., Bedford K., Rissel C., Low E. (2011). Lessons learnt from a pilot bicycle program with community mental health service consumers. Health Promot. J. Austr..

[28] Johnson R., Margolis S. (2013). A review of the effectiveness of adult cycle training in Tower Hamlets, London. Transp. Policy.

[29] Coles E., Wells M., Maxwell M., Harris F.M., Anderson J., Gray N.M., Milner G., MacGillivray S. (2017). The influence of contextual factors on healthcare quality improvement initiatives: What works, for whom and in what setting? Protocol for a realist review. Syst. Rev..

[30] Sallis J.F., Cerin E., Conway T.L., Adams M.A., Frank L.D., Pratt M., Salvo D., Schipperijn J., Smith G., Cain K.L. (2016). Physical activity in relation to urban environments in 14 cities worldwide: A cross-sectional study. Lancet.

[31] Braun L.M., Rodriguez D.A., Cole-Hunter T., Ambros A., Donaire-Gonzalez D., Jerrett M., Mendez M.A., Nieuwenhuijsen M.J., de Nazelle A. (2016). Short-term planning and policy interventions to promote cycling in urban centers: Findings from a commute mode choice analysis in Barcelona, Spain. Transp. Res. Part A Policy Pract..

[32] Wilkie S., Townshend T., Thompson E., Ling J. (2018). Restructuring the built environment to change adult health behaviors: A scoping review integrated with behavior change frameworks. Cities Health.

[33] De Geus B., De Bourdeaudhuij I., Jannes C., Meeusen R. (2008). Psychosocial and environmental factors associated with cycling for transport among a working population. Health Educ. Res..

[34] Bjorkelund O.A., Degerud H., Bere E. (2016). Socio-demographic, personal, environmental and behavioral correlates of different modes of transportation to work among Norwegian parents. Arch. Public Health.

[35] Mertens L., Van Cauwenberg J., Ghekiere A., De Bourdeaudhuij I., Deforche B., Van de Weghe N., Van Dyck D. (2016). Differences in environmental preferences towards cycling for transport among adults: A latent class analysis. BMC Public Health.

[36] Panter J., Jones A., Sluijs E., Griffin S., Wareham N. (2013). Environmental And Psychological Correlates of Older Adult’s Active Commuting. Med. Sci. Sports Exerc..

[37] Buehler R. (2012). Determinants of bicycle commuting in the Washington, DC region: The role of bicycle parking, cyclist showers, and free car parking at work. Transp. Res. D Transp. Environ..

[38] Ma L., Dill J. (2017). Do people’s perceptions of neighborhood bikeability match” reality”?. J. Transp. Land Use.

[39] Assembly G. (2015). United Nations: Transforming our world: The 2030 agenda for sustainable development. Tech. Rep..

[40] Harriss D., Macsween A., Atkinson G. (2017). Standards for Ethics in Sport and Exercise Science Research: 2018 Update. Int. J. Sports Med..

[41] World Medical Association (2013). World Medical Association Declaration of Helsinki: Ethical principles for medical research involving human subjects. JAMA.

[42] Spittaels H., Verloigne M., Gidlow C., Gloanec J., Titze S., Foster C., Oppert J.M., Rutter H., Oja P., Sjöström M. (2010). Measuring physical activity-related environmental factors: Reliability and predictive validity of the European environmental questionnaire ALPHA. Int. J. Behav. Nutr. Phys. Act..

[43] Herrador-Colmenero M., Ruiz J.R., Ortega F.B., Segura-Jiménez V., Álvarez-Gallardo I.C., Camiletti-Moirón D., Estévez-López F., Delgado-Fernández M., Chillón P. (2015). Reliability of the ALPHA environmental questionnaire and its association with physical activity in female fibromyalgia patients: The al-Andalus project. J. Sports Sci..

[44] Chillón P., Ortega F.B., Ruiz J.R., Pérez I.J., Martín-Matillas M., Valtueña J., Gómez-Martínez S., Redondo C., Rey-López J.P., Castillo M.J. (2009). Socio-economic factors and active commuting to school in urban Spanish adolescents: The AVENA study. Eur. J. Public Health.

[45] Garcia-Cervantes L., Martinez-Gomez D., Rodriguez-Romo G., Cabanas-Sanchez V., Marcos A., Veiga O.L. (2014). Reliability and validity of an adapted version of the ALPHA environmental questionnaire on physical activity in Spanish youth. Nutr. Hosp..

[46] Carroll M.D., Curtin L.R., Dohrmann S.M., Hirsch R., Johnson C.L., Kruszan-Moran D., Mirel L.B., Mohadjer L.K., Montaquila J.M., Schober S. (2012). The National Health and Nutrition Examination Survey: Sample Design, 1999–2006. Vital Health Stat. Ser. 2 Data Eval. Methods Res..

[47] Herrador-Colmenero M., Harrison F., Villa-González E., Rodríguez-López C., Ortega F.B., Ruiz J.R., Jones A., Chillón P. (2017). Weather Conditions and Changes in Usual Mode of Commuting to School in Youths. J. Transp. Health.

[48] Field A. (2013). Discovering Statistics Using IBM SPSS Statistics.

[49] Cohen J. (2013). Statistical Power Analysis for the Behavioral Sciences.

[50] Rissel C., Watkins G. (2014). Impact on cycling behavior and weight loss of a national cycling skills program (AustCycle) in Australia 2010–2013. J. Transp. Health.

[51] Zander A., Passmore E., Mason C., Rissel C. (2013). Joy, exercise, enjoyment, getting out: A qualitative study of older people’s experience of cycling in Sydney, Australia. Int. J. Environ. Res. Public Health.

[52] Bopp M., Child S., Campbell M. (2014). Factors associated with active commuting to work among women. Women Health.

[53] Wallace D., Chamberlain A.W., Fahmy C. (2019). Changes in neighborhood social control and disorder and their relationship to exercise behavior. Environ. Behav..

[54] Aittasalo M., Tiilikainen J., Tokola K., Suni J., Sievänen H., Vähä-Ypyä H., Vasankari T., Seimelä T., Metsäpuro P., Foster C. (2019). Socio-Ecological Natural Experiment with Randomized Controlled Trial to Promote Active Commuting to Work: Process Evaluation, Behavioral Impacts, and Changes in the Use and Quality of Walking and Cycling Paths. Int. J. Environ. Res. Public Health.

[55] Aldred R., Jungnickel K. (2014). Why culture matters for transport policy: The case of cycling in the UK. J. Transp. Geogr..

[56] Westman J., Johansson M., Olsson L.E., Mårtensson F., Friman M. (2013). Children’s affective experience of every-day travel. J. Transp. Geogr..

[57] Klinger T., Kenworthy J., Lanzendorf M. (2013). Dimensions of urban mobility cultures—A comparison of German cities. J. Transp. Geogr..

[58] Handy S.L., Wee B., Kroesen M. (2014). Promoting cycling for transport: Research needs and challenges. Transp. Rev..

