# Supplementary material for: Preliminary Results of a Bicycle Training Course on Adults’ Environmental Perceptions and Their Mode of Commuting
Source: Int J Environ Res Public Health. 2022 Mar 15;19(6):3448. doi: 10.3390/ijerph19063448 (PMC8955713; doi:10.3390/ijerph19063448)
Supplement: Supplementary file 1 [file ijerph-19-03448-s001.zip › ijerph-1575932-supplementary.pdf]

International Journal of
Environmental Research and Public Health

MDPI

Supplementary Material

Table S1. Short version of the Assessing Levels of Physical Activity and fitness (ALPHA) questionnaire.

ÍTEMS	STRONGLY DISAGREE	SOMEWHAT DISAGREE	SOMEWHAT AGREE	STRONGLY AGREE	
Most of the houses in my neighborhood are detached houses	**1**	**2**	**3**	**4**	
Many shops to buy things I need are within easy walking distance of my home	**1**	**2**	**3**	**4**	
There is a transit stop within easy walking distance of my home away	**1**	**2**	**3**	**4**	
There are many different routes for cycling or walking from place to place in my neighborhood	**1**	**2**	**3**	**4**	
Walking and cycling are unsafe because of the traffic in my neighborhood	**1**	**2**	**3**	**4**	
Walking and cycling are safe because of the level of crime in my neighborhood	**1**	**2**	**3**	**4**	
My local neighborhood is a pleasant environment for walking and cycling	**1**	**2**	**3**	**4**	
There is an open recreation area within easy walking distance of my home	**1**	**2**	**3**	**4**	
I have access to exercise and sport facilities at work	**1**	**2**	**3**	**4**	NA
My work place provides facilities to support me walking or cycling to work	**1**	**2**	**3**	**4**	NA

Table S2. Mode of commuting questions.

QUESTIONS	WALKING	CYCLING	BY CAR	BY MOTORCYCLE	BY BUS	BY TRAIN	OTHERS
How do you usually commute to local shops?							
How do you usually commute to the supermarket?							
How do you usually commute to local services?							
How do you usually commute to your work place?							
How do you usually commute from your work place?							
